# Optimization of self-microemulsifying drug delivery system for phospholipid complex of telmisartan using D-optimal mixture design

**DOI:** 10.1371/journal.pone.0208339

**Published:** 2018-12-05

**Authors:** Ho Yong Son, Bo Ram Chae, Ji Yeh Choi, Dong Jun Shin, Yoon Tae Goo, Eun Seok Lee, Tae Hoon Kang, Chang Hyun Kim, Ho Yub Yoon, Young Wook Choi

**Affiliations:** 1 College of Pharmacy, Chung-Ang University, Dongjak-gu, Seoul, Republic of Korea; 2 Department of Psychology, National University of Singapore, Singapore, Republic of Singapore; VIT University, INDIA

## Abstract

To improve the dissolution behavior of telmisartan (TMS), a poorly water-soluble angiotensin II receptor blocker, TMS-phospholipid complex (TPC) was prepared by solvent evaporation method and characterized by differential scanning calorimetry and powder X-ray diffractometry. The crystalline structure of TMS was transited into an amorphous state by TPC formation. The equilibrium solubility of TPC (1.3–6.1 mg/mL) in various vehicles was about 100 times higher than that of TMS (0.009–0.058 mg/mL). TPC-loaded self-microemulsifying drug delivery system (SMEDDS) formulation was optimized using the D-optimal mixture design with the composition of 14% Capryol 90 (oil; X_1_), 59.9% tween 80 (surfactant; X_2_), and 26.1% tetraglycol (cosurfactant; X_3_) as independent variables, which resulted in a droplet size of 22.17 nm (Y_1_), TMS solubilization of 4.06 mg/mL (Y_2_), and 99.4% drug release in 15 min (Y_3_) as response factors. The desirability function value was 0.854, indicating the reliability and accuracy of optimization; in addition, good agreement was found between the model prediction and experimental values of Y_1_, Y_2_, and Y_3_. Dissolution of raw TMS was poor and pH-dependent, where it had extremely low dissolution (< 1% for 2 h) in water, pH 4, and pH 6.8 media; however, it showed fast and high dissolution (> 90% in 5 min) in pH 1.2 medium. In contrast, the dissolution of the optimized TPC-loaded SMEDDS was pH-independent and reached over 90% within 5 min in all the media tested. Thus, we suggested that phospholipid complex formation and SMEDDS formulation using the experimental design method might be a promising approach to enhance the dissolution of poorly soluble drugs.

## Introduction

Telmisartan (2-(4-{[4-methyl-6-(1-methyl-1H-1,3-benzodiazol-2-yl)-2-propyl-1H-1,3-benzodiazol-1-yl]methyl}phenyl) benzoic acid; TMS), an angiotensin II receptor blocker, has been widely used for the treatment of hypertension and prevention of strokes over the last decades [1]. TMS has a pKa value of 4.45 and is categorized as a biopharmaceutical classification system (BCS) class II drug, indicating that it is highly permeable and practically insoluble in water [2, 3]. Absorption of TMS from the gastrointestinal (GI) tract is rapid (T_max_ = 0.5–1 h); however, its absolute bioavailability (BA) is relatively low (approximately 43%) because of the limited and pH-dependent solubility [4, 5]. It is practically insoluble in the range of pH 3–9, whereas the solubility increases under strong acidic or basic conditions. To overcome this pH-dependent solubility problem, various approaches, including alkalinization and solubilization, have been used [2].

Solubilization of poorly soluble drugs involve diverse techniques, such as solid dispersion formulation using various types of polymers [6–9], modulation of the microenvironmental pH using various alkalinizers [2, 10], complex formation with phospholipids [11–14], and lipid-based formulation using oils and surfactants [15, 16]. Among those techniques, incorporation of alkalinizing agents has been recognized as one of the most efficient approaches to solubilize TMS. A solid dispersion consisting of polyvinylpyrrolidone K30 and sodium carbonate improved the aqueous solubility and dissolution rate of TMS by approximately 40,000- and 3-fold, respectively [17]. However, modulation of pH using an alkalinizer may be limited by the low stability and toxicity [18, 19]. Also, during mechanical processing or storage, solid dispersion formulation containing a pH modifier may undergo recrystallization leading to the low stability and decreased dissolution rate [20]. Meanwhile, phospholipid complex formation of poorly soluble drugs has been widely investigated to enhance aqueous solubility and/or oral bioavailability [11–14]. Specifically, Zhang et al. [13] demonstrated the beneficial effects of the phospholipid complex on increasing the oil solubility of morin, a poorly water-soluble drug, enabling further development of a lipid-based emulsifying drug delivery system with high drug-payload.

Self-microemulsifying drug delivery system (SMEDDS) has been considered as an alternative approach to enhance the solubility and drug dissolution. It is defined as an isotropic mixture of oil, surfactant, and cosurfactant that rapidly forms a fine oil-in-water (o/w) microemulsion by gentle agitation upon dilution with aqueous medium in the GI tract, extending the interfacial area and drug distribution [21, 22]. Cho et al. [5] formulated a TMS-containing SMEDDS, consisting of glyceryl monooleate (Peceol), caprylocaproyl polyoxyl-8 glycerides (Labrasol), purified diethylene glycol monoethyl ether (Transcutol HP), and triethanolamine; this formulation exhibited a higher dissolution rate than that of raw TMS. To develop an optimized SMEDDS formulation successfully, selection of the components, such as oil, surfactant, and cosurfactant, and well-balanced proportion of the constituents are crucial factors. Experimental design approaches have been widely used in the development of a suitable formulation [23].

Determination of the optimal proportions of SMEDDS components has been empirically performed based on traditional one-factor-at-a-time approaches. However, not only are these methods time-consuming, labor-intensive, and inefficient, they also often provide inadequate data to analyze the effect of each component and their potential interactions [5, 24]. Thus, statistical optimization approaches have been introduced to estimate the effects of mixture-related factors and the interaction between multicomponents of independent variables [25–27]. Recently, a statistical optimization tool based on response surface methodology and experimental designs such as central composite, Box–Behnken, factorial, and mixture designs has been introduced. The D-optimal mixture design is one of the most popular response surface methodologies for optimizing SMEDDS formulation, because it minimizes the variance associated with the evaluation of coefficients in a model and produces the best-possible subset by considering the criteria for maximizing information matrix determinants [28]. In addition, the D-optimal mixture design considers the total system of SMEDDS as 100%, while the central composite, Box–Behnken, and factorial designs do not consider the total system of SMEDDS formulation [29].

In this study, TMS-phospholipid complex (TPC) was prepared to facilitate the incorporation of TMS into a SMEDDS formulation, and thus, to overcome the pH-dependent solubility problem of TMS. Crystalline property of TPC was characterized by differential scanning calorimetry (DSC) and powder X-ray diffractometry (PXRD). TPC-loaded SMEDDS formulation was optimized using the D-optimal mixture design with the composition of Capryol 90 (oil; X_1_), tween 80 (surfactant; X_2_), and tetraglycol (cosurfactant; X_3_) as independent variables, and the observation of a droplet size (Y_1_), TMS solubilization (Y_2_), and drug release in 15 min (Y_3_) as response factors. And the pH-independent dissolution profiles of the optimized TPC-loaded SMEDDS formulation were further evaluated.

## Materials and methods

### Materials

TMS was supplied by Daewon Pharmaceutical Co., Ltd (Seoul, Korea). Soy phosphatidylcholine (SPC; 95%) was purchased from Avanti Polar Lipids (Alabaster, AL, USA). Capmul MCM was purchased from Abitec Co. (Janesville, WI, USA). Tween 80 and tetraglycol were purchased from Sigma-Aldrich (St. Louis, MO, USA). Capryol 90 and Transcutol P were purchased from Gattefosse (Saint-Priest, France). Cremophor RH 40 was purchased from BASF (Ludwigshafen, Germany). High-performance liquid chromatography (HPLC) grade methanol was purchased from J.T. Baker (Phillipsburg, NJ, USA). All other chemicals used were of analytical grade.

### Preparation of TPC

TPC was prepared using TMS and SPC at different molar ratio. Briefly, accurately weighed amounts of TMS (150 mg) and SPC (150, 225, and 300 mg for 1.5:1, 1:1, and 1:1.5 molar ratio of TMS:SPC, respectively) were added to dichloromethane (30 mL) in a 100-mL round bottom flask. The mixture was agitated using a magnetic stirrer (IKA, Staufen, Germany) at 25°C until a clear solution was obtained, and then the solvent was evaporated using a vacuum rotary evaporator (N-1300, EYELA, Tokyo, Japan). The remaining white residues were collected as TPC and stored in the freezer at -20°C. The samples were used for the experiment within 4 weeks and no changes were observed until use.

### Characterization of TPC

Solid-state properties of TPC, TMS, SPC, and physical mixtures were investigated using DSC and PXRD. The DSC thermograms of samples were obtained by using DSC-Q20 (TA instrument, New Castle, DE, USA). Each sample (3–5 mg) was placed in an aluminum pan and heated at a rate of 5°C/min for a temperature range of 0–300°C under nitrogen flow (20 mL/min). The PXRD patterns of samples were verified using an X-ray diffractometer (D8 Advance, Bruker, Germany) with Nickel-filtered Cu Kα radiation. The X-ray diffractogram was scanned at the 2θ range of 5–60° with a scanning speed of 5°/min, and the step angle was 0.02°.

### Solubility measurement

The solubility of TMS and TPC in various vehicles was determined by the equilibrium method and expressed as the equivalent concentration of TMS solubilized. Briefly, an excess amount of TMS or TPC was added to 1 mL of the selected vehicles of SMEDDS formulation or its components. Test tubes containing the mixtures were sealed and kept at 25°C with intermittent shaking (CM-1000, EYELA, Tokyo, Japan) for 24 h to reach equilibrium. The mixtures were then centrifuged (Smart R17, Hanil Science Industrial, Incheon, Korea) at 14,000 ×*g* for 10 min to remove the undissolved TMS or TPC. The supernatant was filtered through a 0.45-μm polyvinylidene difluoride **(**PVDF) membrane filter (Whatman International Ltd., Kent, UK), and the concentration of TMS in the filtrate was measured using HPLC after appropriate dilution with methanol.

### HPLC analysis of TMS

TMS concentration was determined using HPLC as reported earlier [30]. The HPLC system consisted of a pump (W2690/5; Waters Corporation, Milford, MA, USA), an ultraviolet detector (W2489; Waters Corporation, Milford, MA, USA), and data station (Empower 3; Waters Corporation). Chromatographic separation was performed using a C18 column (Agilent TC-C18, 4.6 × 150 mm, 5 μm; Agilent Technologies, Palo Alto, CA, USA) at a wavelength of 298 nm and a flow rate of 1 mL/min at 25°C. The isocratic mobile phase was composed of methanol and ammonium dihydrogen phosphate buffer (70:30, v/v), and the pH was adjusted by adding 10% hydrochloric acid. A certain volume (20 μL) of each sample was injected, and TMS concentration was calculated from the calibration curve, in which the linearity of the least-square linear regression was established in the range of 1–100 μg/mL with a coefficient of determination (r^2^) value > 0.99.

### Formulation of SMEDDS

The microemulsion regions were determined using a pseudoternary phase diagram, composed of oil, surfactant, and cosurfactant, where each component was indicated at the apex of a triangle. Based on the results of previous studies [27, 29], several vehicles were screened, including Capryol 90 and Capmul MCM as an oil; Cremophor RH 140 and tween 80 as a surfactant; tetragycol and Transcutol P as a cosurfactant. Considering the solubility and microemulsion boundary, Capryol 90, tween 80, and tetraglycol were finally selected, and a series of blank SMEDDS formulations was prepared for each of the three components at various concentrations. For any mixture, the sum of the concentrations of the three components was always 100%. Prepared SMEDDS samples were stored in ambient condition at 25°C and used for the experiment within 4 weeks. Until use, any appreciable changes in either physical appearance or drug contents were not observed.

### Determination of droplet size

A dynamic light scattering particle size analyzer (Zetasizer Nano-ZS, Marlvern Instrument, Worcestershire, UK) was used to measure the particle size of emulsion droplets. Aliquots (10 μL) of each TPC-free SMEDDS formulation were diluted with 10 mL of distilled water and gently vortexed to obtain a homogenous dispersion. The samples were loaded into a disposable cuvette placed in a thermostatic chamber at 25°C, and light scattering was monitored using a 50 mV laser at an angle of 90°.

### Experimental design for optimizing TPC-loaded SMEDDS

The D-optimal mixture design was used to optimize the composition of TPC-loaded SMEDDS formulation using the Minitab software (ver. 17.0; Minitab Inc, State College, PA, USA). The experiment was designed using the three components as independent variables. Based on the results of the pseudoternary phase diagram, concentrations of Capryol 90 (oil; X_1_), tween 80 (surfactant; X_2_), and tetraglycol (cosurfactant; X_3_) were set within the ranges of 10–30%, 30–70%, and 20–60%, respectively. For any experiment, the sum of the concentrations of X_1_, X_2_, and X_3_ was 100%. Droplet size (Y_1_; nm), TMS solubilization (Y_2_; mg/mL), and percentage of drug released in 15 min (Y_3_; %) were evaluated as the response variables to determine the optimal TPC-loaded SMEDDS formulation with a high desirability value. The design consisted of nine experimental points to find a model fit, evaluate the effects of independent variables on the responses, and estimate the experimental error in the responses.

### *In vitro* dissolution test

Dissolution tests were performed according to the USP apparatus II (paddle) method using a Vision Classic 6 dissolution tester and a Vision heater (Hanson, Chatsworth, CA, USA) at 37 ± 0.5°C. The revolution speed of paddle and volume of dissolution medium were 50 rpm and 900 mL, respectively. Distilled water and solutions of pH 1.2, 4, and 6.8 were used as the dissolution media. As soon as the paddles were rotated, the test formulation equivalent to 5 mg of TMS was introduced into the dissolution medium. Samples (5 mL) were obtained at predetermined time points (5, 15, 30, 60, 90, and 120 min) and filtered through a 0.45-μm PVDF membrane filter. The amount of dissolved TMS in the filtrate was measured using HPLC after appropriate dilution with methanol.

## Results and discussion

### Solubility of TMS and TPC

The solubility of drugs in SMEDDS formulation is one of the most important factors in the development of drug-loaded SMEDDS formulations. In the preliminary screening of the oil component, Capryol 90 and Capmul MCM were selected due to the relatively higher TMS solubility (8.7–9.7 μg/mL) compared with other oils tested, such as olive oil (0.06 μg/mL), corn oil (0.11 μg/mL), Laurogylcol 90 (3.87 μg/mL), and Labrafil 1944 (2.41 μg/mL). Table 1 presents the equilibrium solubility of TMS and TPCs in the selected vehicles, which have been used as potential components of SMEDDS formulations in previous studies [27, 29]. Overall, in the majority of the vehicles tested, the solubility of TPCs was about 100 times higher than that of TMS (solubility ranges of 1.3–6.1 mg/mL (TPCs) *versus* 0.009–0.058 mg/mL (TMS)). There was no significant difference between TPCs prepared with different molar ratio of TMS:SPC. However, TPC of 1:1 molar ratio was somewhat superior to others, subsequently being selected as a representative TPC for further studies. Phospholipid complex formation generally increases the solubility of poorly soluble drugs [12–14]. Maiti et al. [12] reported the enhanced solubility of curcumin-phospholipid complex in water and *n*-octanol (3.2-fold and 11.3-fold, respectively), compared to that of raw curcumin. Zhang et al. showed that the solubility of morin-phospholipid complex in various oils and distilled water was significantly higher (3.9- to 6.5-fold and 4-fold, respectively) than that of the raw drug molecule [13]. In particular, tween 80 and tetraglycol, used as a surfactant and cosurfactant, respectively, in our experiment, significantly increased the solubility of TPCs; thus, they were selected as SMEDDS components. However, the solubility of TPCs in oil was lower than that in other components, showing an insignificant difference between Capryol 90 and Capmul MCM. Thus, both oils were further compared by the ternary phase diagram study.

**Table 1:** Equilibrium solubility of TMS and TPCs in the selected vehicles.

Vehicle	TMS (μg/mL)	TPCs (mg/mL)*		
		1.5:1	1:1	1:1.5
*Oil*				
Capryol 90	8.76 ± 1.36	1.38 ± 0.06	1.32 ± 0.03	1.56 ± 0.07
Capmul MCM	9.73 ± 1.00	1.60 ± 0.04	1.60 ± 0.04	1.53 ± 0.11
*Surfactant*				
Cremophor RH40	57.76 ± 1.49	2.01 ± 0.19	4.58 ± 1.29	1.10 ± 0.13
Tween 80	41.42 ± 3.67	4.69 ± 0.21	6.14 ± 0.60	4.99 ± 0.18
*Cosurfactant*				
Tetraglycol	24.29 ± 0.28	3.91 ± 0.13	4.16 ± 0.10	4.11 ± 0.14
Transcutol P	34.02 ± 2.04	2.34 ± 0.11	2.39 ± 0.07	2.36 ± 0.03

* Prepared at different molar ratio of TMS:SPC.

The solubility was expressed as the equivalent concentration of TMS solubilized.

Data are presented as the mean ± standard deviation (*n* = 3).

TMS, telmisartan; TPC, telmisartan-phospholipid complex; SPC, Soy phosphatidylcholine

### Characterization of TPC

**Fig 1:**
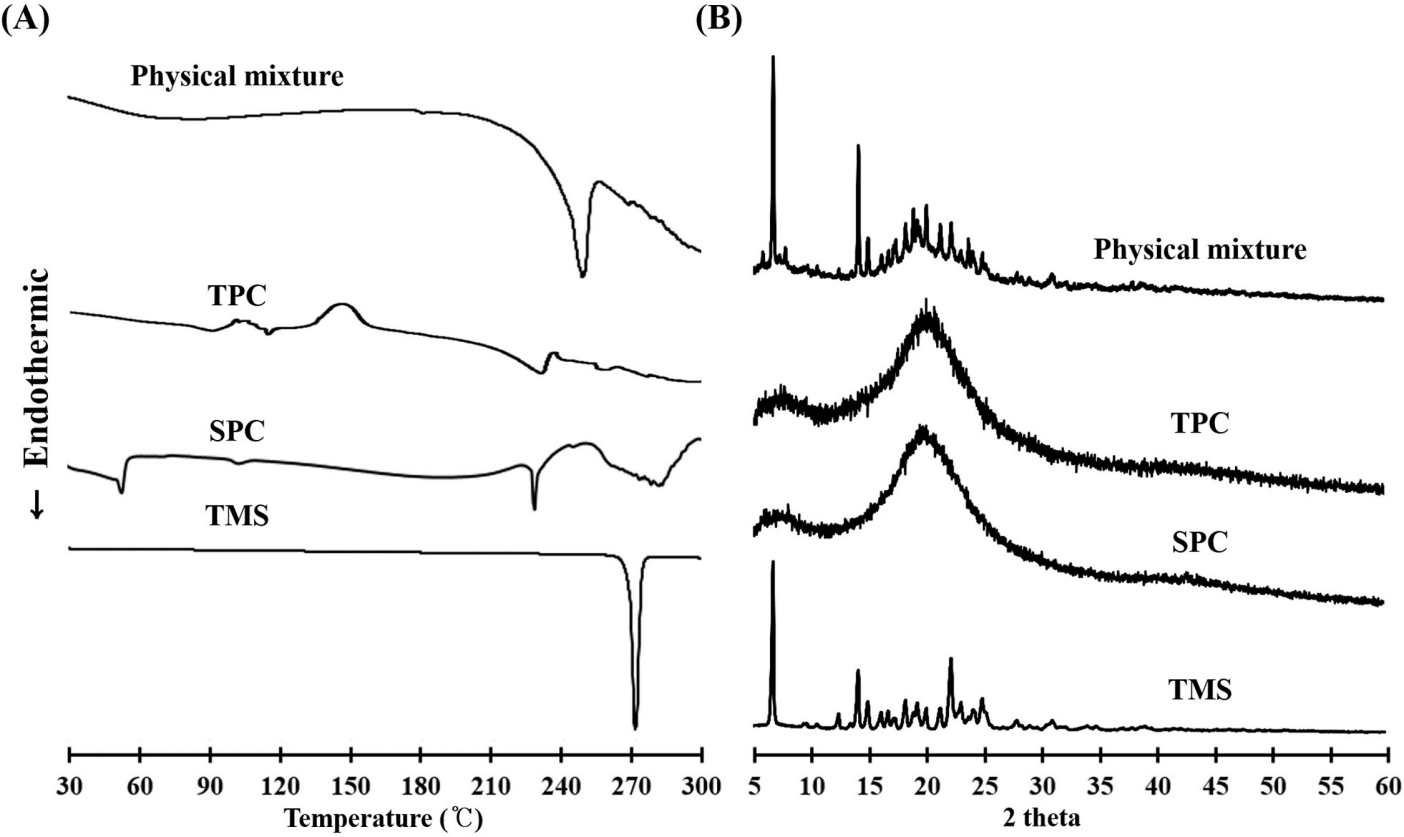
Solid-state properties of different samples: Telmisartan (TMS), soy phosphatidylcholine (SPC), TMS-PC complex (TPC), and the physical mixture of TMS and SPC. **(A)** Differential scanning calorimetric thermograms. **(B)** Powder X-ray diffraction patterns.

To evaluate the effects of complex formation on the crystalline structure of the active pharmaceutical ingredient, different samples (raw TMS, SPC, TPC, and a physical mixture of TMS and SPC) were subjected to DSC and PXRD analyses. DSC thermograms are shown in Fig 1A. Thermal analysis can provide information related to melting, recrystallization, decomposition, and change in specific heat capacity that determines the physicochemical properties of a compound [17]. The DSC curve for raw TMS powder exhibited a sharp endothermic peak at 269.1°C, which corresponded to its intrinsic melting point (261–263°C) [31]. However, no melting peak of TMS was identified in TPC, indicating that TMS existed in a non-crystalline state. SPC showed two different endothermic peaks, which were consistent with previous studies, showing that the first mild endothermic peak at 53.6°C could be attributed to hot movements of the polar parts of the phospholipid molecule, and the second endothermic peak at 228.3°C might result from the transition from the gel state to liquid crystalline state and the melting of carbon-hydrogen chain in phospholipids [32, 33]. The physical mixture of TMS and SPC showed different peak patterns; a weak shift in the baseline around 60–80°C, which was close to the first melting peak of SPC, and a sharp peak at 249.5°C, which possibly originated from the TMS peak, although the melting range was somewhat lowered. PXRD patterns of TMS powder, SPC, TPC, and the physical mixture are presented in Fig 1B. The diffraction pattern of TMS showed numerous intrinsic peaks at various angles up to 30°, indicating its crystalline nature. In contrast, although the inherent broad peak of SPC was present, no intrinsic peaks were observed with TPC, indicating the presence of TMS in the amorphous state in the phospholipid complex. The physical mixture showed all the major characteristic peaks of raw TMS powder and SPC at various diffraction angles, indicating the crystallinity of the drug in the mixture. These results suggested that the internal structural transition of TMS into an amorphous state was induced by TPC formation.

Phospholipid complex formation between TMS and SPC might occur by noncovalent interactions, including van der Waals forces and/or hydrogen bonding. Numerous studies reported the molecular interactions of phospholipids with therapeutic drugs. In the liposomal encapsulation of indomethacin, the negative carboxylate groups of the drug would preferably stay in the region of the positive trialkylamino groups of phosphatidylcholine [11]. Curcumin-phospholipid complex was previously prepared, in which the hydroxyl groups of the phenol rings of curcumin were involved in hydrogen bonding, whereas the aromatic rings were involved in hydrophobic interactions [12]. In morin-phospholipid complex-loaded SMEDDS formulation, morin molecule was entrapped in the polar head group of phospholipid molecules; thus, the crystalline characteristics of the entrapped molecule were lost [13].

### Ternary phase diagram for SMEDDS formulation

**Fig 2:**
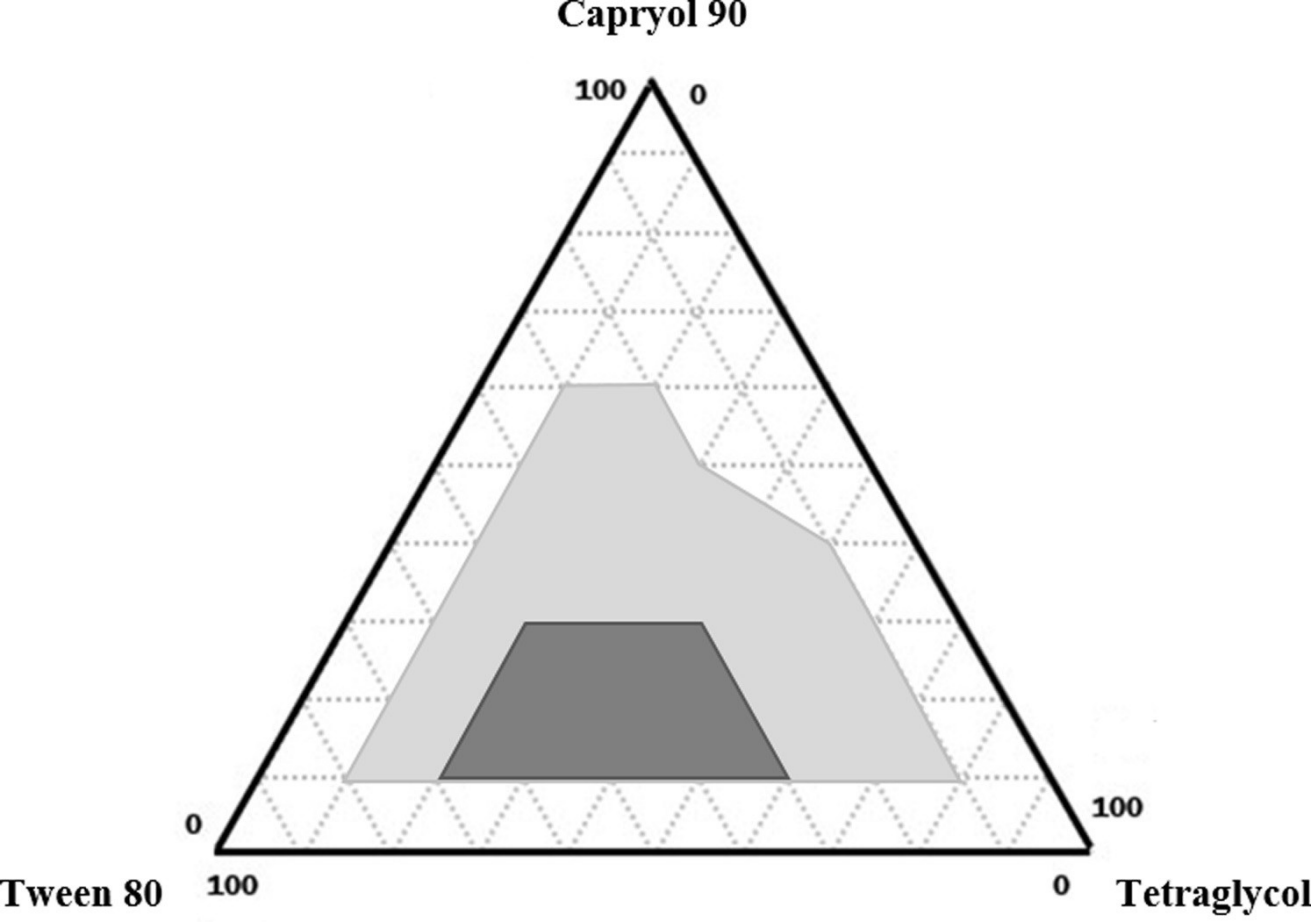
Ternary phase diagram of Capryol 90 (oil), tween 80 (surfactant), and tetraglycol (cosurfactant). Light gray and dark gray areas indicate self-microemulsifying regions and experimental regions, respectively.

After the selection of surfactant (tween 80) and cosurfactant (tetraglycol), both Capryol 90 and Capmul MCM were used to construct a ternary phase diagram. Selection of the oil component is a crucial step for SMEDDS formulation because it acts as a main excipient for solubilizing the hydrophobic drug molecules. In the preliminary study with both oils, Capryol 90-based SMEDDS formulation showed a larger area of self-microemulsifying region than that of Capmul MCM-based SMEDDS formulation (S1 Fig). Thus, Capryol 90 was finally selected for further evaluation of the droplet size and polydispersity index (PDI) (S1 Table). As shown in Fig 2, the light gray area represents the stable microemulsification region with a droplet size < 200 nm. In particular, the SMEDDS region representing a PDI value < 0.3 was designated with dark gray at a boundary of 10–30% Capryol 90, 30–70% tween 80, and 20–60% tetraglycol, and was considered the testing range for the experimental design study.

### Statistical analysis using D-optimal mixture design

To optimize the TPC-loaded SMEDDS composition, a mixture design method was employed using Minitab software (Minitab Inc, State College, PA, USA). In this method, responses were assumed to depend on the proportions of the excipients present in the mixture. Based on the experimental region of the ternary phase diagram, Capmul MCM, tween 80, and tetraglycol were selected as independent variables (X_1_, X_2_, and X_3_, respectively; Table 2). Three response factors were selected: the droplet size (nm; Y_1_), degree of TMS solubilization (mg/mL; Y_2_), and percentage of drug released in 15 min (%; Y_3_). As shown in Table 3, nine experimental runs were determined according to the mixture design. Y_1_ ranged from 15.2 to 169.2 nm, Y_2_ from 3.13 to 4.15 mg/mL, and Y_3_ from 69.7 to 98.7%. All three responses were fitted to linear, quadratic, special cubic, and full cubic models. The results of goodness-of-fit statistical measures for the four models are shown in Table 4. The standard error (SE) of regression indicated the average vertical distance of data values from the fitted regression line. The predicted residual error sum of squares (PRESS) was used to determine how well a given model fits the data, where the smaller the value, the better the predictive ability of the model. R^2^ represents the variation in a response variable owing to all the independent variables in a given model. A higher R^2^ indicates that the model explains all the variability of the response variable. Based on the values of SE, PRESS, and R^2^, the full cubic model was considered the best fitted model for each of the three responses. Therefore, full cubic models were selected for further optimization.

**Table 2:** Variables and responses used in the D-optimal mixture design.

Independent variables		Range (%)	
		Minimum	Maximum
X_1_	Capyol 90 (%; oil)7	10	30
X_2_	Tween 80 (%; surfactant)	30	70
X_3_	Tetraglycol (%; cosurfactant)	20	60
Responses		Goal	
Y_1_	Droplet size (nm)	Minimize	
Y_2_	TMS solubilization (mg/mL)	Maximize	
Y_3_	DR_15_ (%)	Maximize	

DR_15_, the percentage of drug released in 15 min.

**Table 3:** Mixture design for optimization of TPC-loaded SMEDDS formulation and the associated response data.

Mixture number	Capryol 90 (%; X_1_)	Tween 80 (%; X_2_)	Tetraglycol (%; X_3_)	Droplet size (nm; Y_1_)	TMS solubilization (mg/mL; Y_2_)	DR_15_ (%; Y_3_)
1	30	50	20	136.6 ± 4.2	3.72 ± 0.08	94.1 ± 3.7
2	25	37.5	37.5	151.7 ± 8.9	3.13 ± 0.13	84.5 ± 5.7
3	15	57.5	27.5	36.1 ± 0.2	4.15 ± 0.09	96.5 ± 1.5
4	30	30	40	169.2 ± 2.5	3.21 ± 0.07	70.5 ± 0.2
5	25	47.5	27.5	151.3 ± 2.8	3.79 ± 0.17	71 ± 2.3
6	10	30	60	106.4 ± 22.6	3.76 ± 0.26	82.8 ± 1.9
7	20	45	35	155.4 ± 1.7	3.81 ± 0.18	79.8 ± 3
8	10	70	20	15.2 ± 0.1	3.93 ± 0.08	69.7 ± 1.1
9	15	37.5	47.5	151.1 ± 7.6	3.65 ± 0.16	98.7 ± 0.1

DR_15_, the percentage of drug released in 15 min.

**Table 4:** Summary of results of statistical analysis and model equations for the measured responses.

Models	R^2^ ^a^	R^2^ (adj)^b^		SE^c^	PRESS^d^	Remark
Droplet size (nm; Y_1_)						
Linear	79.93	78.26	24.75		17996.4	
Quadratic	86.53	83.33	21.67		15288	
Special cubic	88.54	85.11	20.49		13960.5	
Full cubic	98.13	97.3	8.72		3077.3	Suggested
TMS solubilization (mg/mL; Y_2_)						
Linear	55.08	51.34	0.233896		1.68881	
Quadratic	76.73	71.19	0.179977		1.09605	
Special cubic	77.7	71.01	0.180531		1.14689	
Full cubic	89.39	84.68	0.131233		0.6975	Suggested
Percentage of drug released in 15 minutes (%; Y_3_)						
Linear	2.08	0	11.4817		4090.59	
Quadratic	70.59	63.59	6.72686		1398.17	
Special cubic	77.87	71.23	5.97921		1170.12	
Full cubic	96.83	95.42	2.38522		230.415	Suggested

^a^Percentage of response variable variation; the higher the value, the better the model fits the data.

^b^Percentage of response variable variation based on its relationship with one or more predictor variables

^c^Standard error of the regression, represents the standard distance between the data values and the fitted regression line.

^d^Prediction error sum of squares, the smaller the PRESS value, the better the model predictive ability.

### Effects of independent variables on the responses in experimental design

Table 5 shows the results of analysis of variances for the three response variables. For Y_1_, the F-test for the linear effect parameters (linear parameters in the full cubic model) indicated significant difference (F(2,18) = 28.8, *p* < 0.001), suggesting that at least one regression coefficient for the linear parameters was significantly different from zero. The statistical significance remained the same for the other two response variables (F(2,18) = 4.5, *p* < 0.027 for Y_2_ and F(2,18) = 46.6, *p* < 0.001 for Y_3_), indicating that at least one independent variable resulted in a significant effect on Y_2_ and Y_3_. The remaining polynomial effect parameters were found to be statistically significant as well. As shown in Table 5, the *p*-values of both linear and polynomial effect parameters were less than 0.05, indicating that they were statistically significant within the model. Accordingly, the final polynomial regression equations were computed as follows:

$$Y_{1}\;=\;66968\;X_{1}\;+\;3672\;X_{2}\;-\;4788\;X_{3}\;-\;138534\;X_{1}X_{2}\;-\;117422\;X_{1}X_{3}+\;4446\;X_{2}X_{3}\;+\;180459\;X_{1}X_{2}X_{3}\;-\;72956\;X_{1}X_{2}\left(X_{1}-X_{2}\right)\;-\;35114\;X_{2}X_{3}\left(X_{2}-X_{3}\right)$$ (1)

$$Y_{2}\;=\;485\;X_{1}\;+\;14.3\;X_{2}\;-\;20.2\;X_{3}\;-\;975.4\;X_{1}X_{2}-\;812.2\;X_{1}X_{3}\;+\;39.3\;X_{2}X_{3}\;+\;1101.8\;X_{1}X_{2}X_{3}\;-\;629.6\;\text{X}1\text{X}2\left(X_{1}-X_{2}\right)\;-\;191.7\;X_{2}X_{3}\left(X_{2}-X_{3}\right)$$ (2)

$$Y_{3}\;=\;-\;27714\;X_{1}\;-\;1410\;X_{2}\;+\;1063\;X_{3}\;+\;59622\;X_{1}X_{2}\;+\;48944\;X_{1}X_{3}\;+\;1746\;X_{2}X_{3}\;-\;80731\;X_{1}X_{2}X_{3}\;+\;37753\;X_{1}X_{2}\left(X_{1}-X_{2}\right)\;+\;13344\;X_{2}X_{3}\left(X_{2}-X_{3}\right)$$ (3)

**Table 5:** Analysis of variance for full cubic model of the measured responses.

Source	DF	Y_1_ (Droplet size; nm)			Y_2_ (TMS solubilization; mg/mL)			Y_3_ (DR_15_, %)		
		Adj SS	F	*P*-value	Adj SS	F	*P*-value	Adj SS	F	*P*-value
Model	8	71895	118.3	< 0.001	2.6131	19	< 0.001	3128.7	68.7	< 0.001
linear	2	4383	28.8	< 0.001	0.1539	4.5	0.027	529.8	46.6	< 0.001
X1X2	1	3091	40.7	< 0.001	0.1532	8.9	0.008	57.5	100.6	< 0.001
X1X3	1	2383	31.4	< 0.001	0.114	6.6	0.019	414	72.8	< 0.001
X2X3	1	1431	18.8	< 0.001	0.1121	6.5	0.02	220.8	38.8	< 0.001
X1X2X3	1	3095	40.7	< 0.001	0.1154	6.7	0.019	619.4	108.9	< 0.001
X1X2(-)	1	1993	26.2	< 0.001	0.1484	8.6	0.009	533.7	93.8	< 0.001
X2X3(-)	1	3133	41.2	< 0.001	0.0934	5.4	0.032	452.5	79.5	< 0.001
residual	18	1368	-	-	0.31	-	-	102.4	-	-

X_1_; oil (Capryol 90), X_2_; surfactant (tween 80), X_3_; cosurfactant (tetraglycol).

DF, degrees of freedom; SS, sum of squares; DR_15_, the percentage of drug released in 15 min.

**Fig 3:**
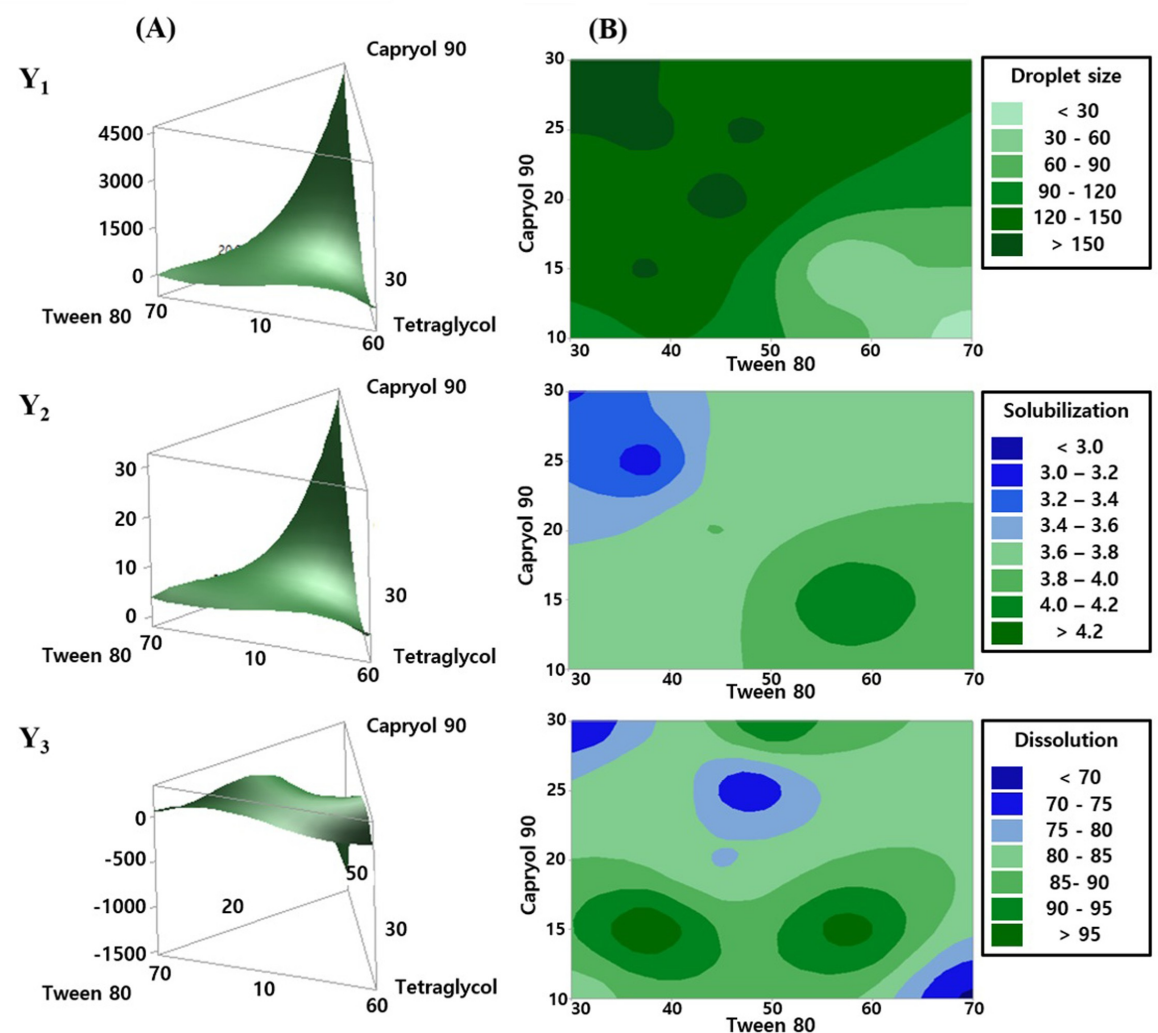
Effects of independent variables on the response factors, Y_1,_ Y_2_, and Y_3_. **(A)** Three-dimensional response surface plot for the effects of the independent variables. **(B)** Contour plots for the effects of X_1_ and X_2_ on the responses. **(**Y_1_, droplet size (nm); Y_2_, TMS solubilization (mg/mL); and Y_3_, the percentage of drug released in 15 min (%)).

The magnitude of each estimated regression coefficient indicated the relative contribution of the corresponding independent variable to the response variable. Since our main objective was to evaluate the combined effects of the three independent variables and to identify their optimal composition, we focused on interpreting the magnitude of the linear effect parameters. In all polynomial equations, it was shown that X_1_ would be the most important and critical independent variable affecting Y_1_, Y_2_, and Y_3_ in the SMEDDS formulation. As shown in the response surface and contour plots in Fig 3, the level of change in the response variables was largely affected by X_1_. A positive effect of X_1_ on Y_1_ in Eq (1) suggested a decrease in Y_1_ with the decrease in X_1_. In particular, when the value of X_1_ was approximately < 15%, the value of Y_1_ decreased (Fig 3B). It is noteworthy that the total sum of the proportions of the three independent variables would amount to 100%. For example, when X_1_ and X_2_ were 15 and 55%, respectively, the corresponding value of X_3_ became 30%.

For the degree of TMS solubilization (Y_2_), Eq (2) and Fig 3A showed that X_1_ had a positive relationship with Y_2_. However, when their relationship was further examined within the actual experimental domain, the contour plot showed a negative relationship between X_1_ and Y_2_. When SMEDDS formulation was composed of approximately 15% X_1_, 60% X_2_, and 25% X_3,_ the highest Y_2_ value was obtained. For the percentage of drug released in 15 min (Y_3_), Eq (3) and Fig 3A indicated that X_1_ had a negative relationship with Y_3_. Similarly, the corresponding contour plot showed that the highest dissolution rate was achieved when X_1_ was approximately 15%. Combining all results of the three response variables (Fig 3B), the composition of SMEDDS was more favorable with 15% X_1_, 60% X_2_, and 25% X_3_.

### Optimization of TPC-loaded SMEDDS using desirability function

Three features are needed for optimization: a limit value (upper or lower), an allotted target value, and an input indicating whether each response should be maximized or minimized. When an optimization process is coupled with maximization, a value for the lower bound is necessary. In contrast, when a response variable is set to be minimized, a value for the upper bound is required. Y_1_ was set to be minimized because the smaller the particle size of SMEDDS formulation, the better the GI absorption [25, 34]. Several studies on SMEDDS and microemulsions suggested that the ideal diameter of a stable microemulsion should be < 200 nm [35, 36]. In our experimental run of mixture design, the minimum experimental value of droplet size was 15.2 nm (Table 3). Thus, both the target value and upper bound were 15 and 200 nm, respectively, for the optimization. Y_2_ was set to be maximized, and a target value and lower bound of 6 and 1 mg/mL, respectively, were selected. The target value of 6 mg/mL was selected based on the highest solubility of TPC in tween 80 (Table 1). The lower bound of 1 mg/mL was chosen based on the solubility of TPC in Capryol 90 (Table 1). Y_3_ was set to be maximized, and a target value and limit value of 100 and 60%, respectively, were selected. The lower bound of 60% was selected based on the lowest percentage of drug released from TPC in 15 min in pH 6.8 medium. All ranges for the response variables were among the values of our experimental run of mixture design.

**Fig 4:**
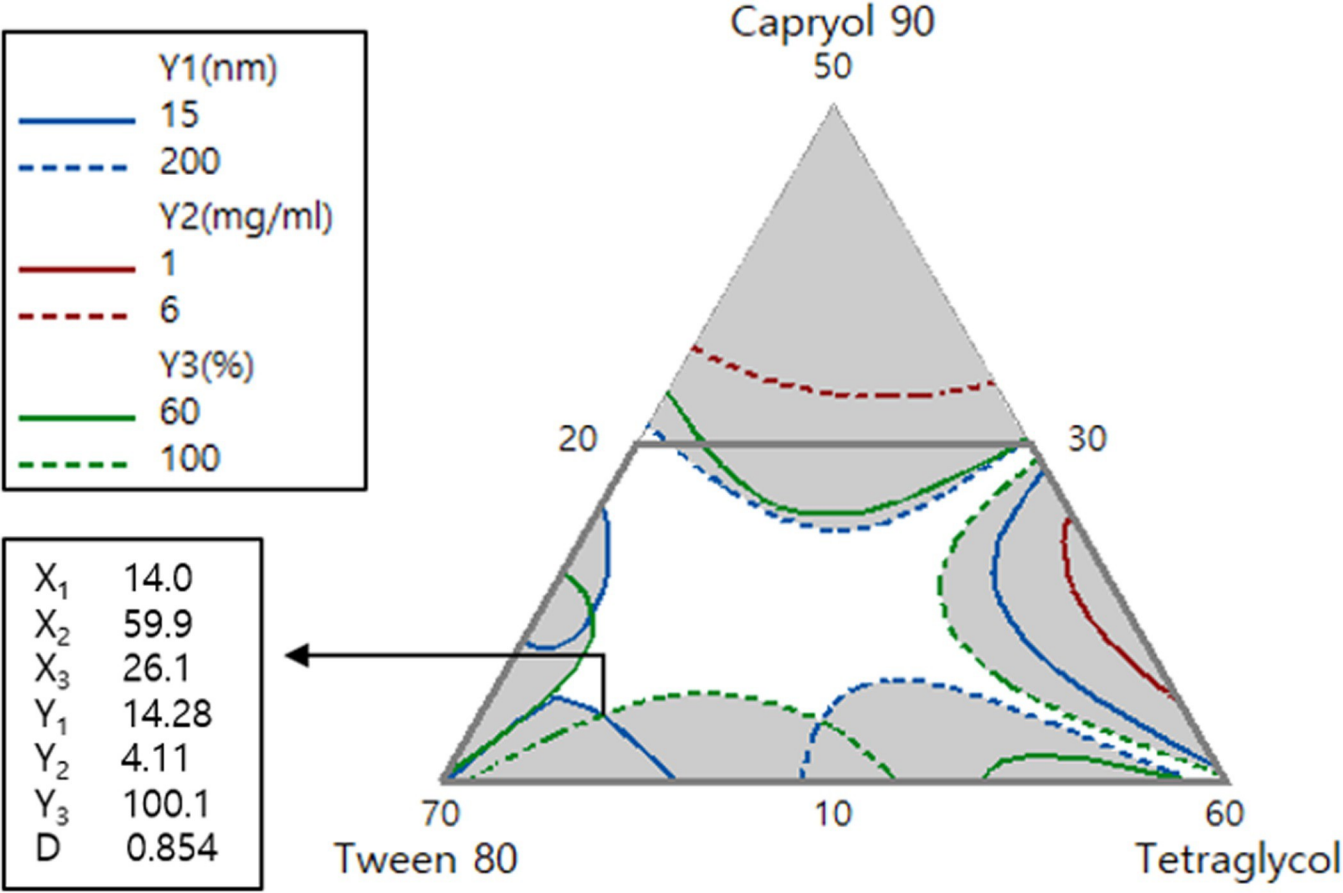
Overlay contour plot of the optimized TPC-loaded SMEDDS formulation. Upper box shows the target and limit values for optimization. Values in the lower box represent the percentage of three components and the predicted responses for the optimized formulation with a desirability value.

The independent variables were optimized for the response values by using the desirability function. Fig 4 represents the overlay plot for the effects of the three independent variables on all response variables. The optimized formulation ratios of TPC-loaded SMEDDS of X_1_, X_2_, and X_3_ were 14, 59.9, and 26.1%, respectively. These values were supported by a desirability function value of 0.854. The predicted and observed values of Y_1_, Y_2_, and Y_3_ for the optimized TPC-loaded SMEDDS formulation are shown in Table 6. Values of prediction errors were calculated to evaluate the reliability and accuracy. Although the prediction error of Y_1_ was relatively high, its variable attained a size that seemed to be suitable considering the experimental range of SMEDDS formulation. The prediction errors of both Y_2_ and Y_3_ were very small and desirable. Therefore, these results indicated that the D-optimal mixture design method used for optimizing TPC-loaded SMEDDS in this study was reliable and accurate. For the percentage of drug released in 15 min (Y_3_), the optimized TPC-loaded SMEDDS showed Y_3_ value of 99.4%. In addition, it exhibited desirable values of droplet size (Y_1_ = 22.17 nm) and TMS solubilization (Y_2_ = 4.06 mg/mL). Thus, this optimized formulation was subjected to further *in vitro* dissolution studies.

**Table 6:** Predicted and observed values of optimized TPC-loaded SMEDDS formulation.

	Droplet size (nm; Y_1_)	TMS solubilization (mg/mL; Y_2_)	DR_15_ (%; Y_3_)
Predicted value	14.28	4.11	100.1
Observed value	22.17	4.06	99.4
Prediction error (%)*	35.6	-1.2	-0.7

*Calculated using the formula ([observed value—predicted value]/observed value) × 100 (%)

DR_15_, the percentage of drug released in 15 min.

### pH-independent dissolution of the optimized TPC-loaded SMEDDS

**Fig 5:**
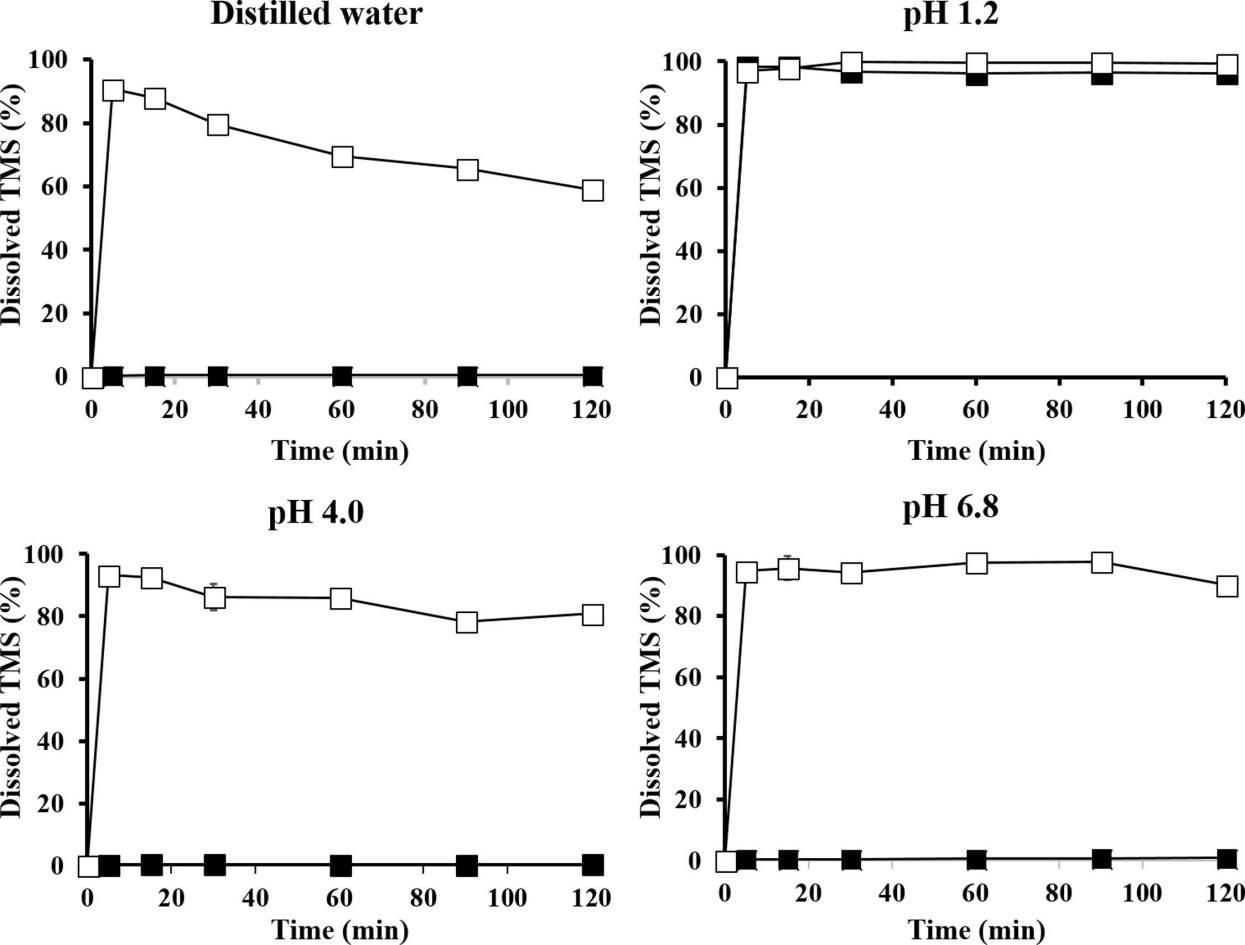
Dissolution profiles of raw TMS (■) and optimized TPC-loaded SMEDDS formulation (□) in different media of distilled water, pH 1.2, pH 4, and pH 6.8. Values represent the mean ± SD (*n* = 3).

The *in vitro* dissolution profiles of raw TMS and the optimized TPC-loaded SMEDDS formulation were determined in various dissolution media, including distilled water, pH 1.2, pH 4, and pH 6.8 (Fig 5), to mimic the different acidity in the GI tract. Dissolution of raw TMS was poor and pH-dependent, where it was extremely low (< 1% for 2 h) in water, pH 4, and pH 6.8 media, whereas it had fast and high dissolution (> 90% in 5 min) in pH 1.2 medium. These results are consistent with previous reports, showing that TMS showed pH-dependent solubility, which was high in strongly acidic and basic conditions (almost 100% dissolution in gastric fluid within 20 min); however, it was extremely low in neutral conditions resulting in low dissolution rate (< 1% for 90 min) in pH 6.8 medium [2, 4].

In contrast, the optimized TPC-loaded SMEDDS formulation showed a pH-independent TMS dissolution profile regardless of the type of dissolution media, where it reached over 90% in 5 min. However, a mild decrease or fluctuation in dissolution was observed at pH 1.2. Overall, the optimized system resulted in a remarkably enhanced dissolution, compared to that of the raw TMS. This improved dissolution was probably attributed to the following reasons. First, phospholipid complexation resulted in a change from the crystalline structure to an amorphous form that is thermodynamically unstable and provides an easy solid-to-liquid phase transition. It is widely known that the conversion of drug structure from the crystalline to amorphous state increases its dissolution rate owing to the high disorder and high energy state of the amorphous form [37]. Similar trends have been observed in polymeric solid dispersions of TMS. Amorphous solid dispersions of TMS, prepared using various polymers, such as polyvinyl pyrrolidone, polyethylene glycol, and poloxamer, by the solvent evaporation method, exhibited a significantly enhanced drug release, compared to that of the raw TMS, possibly owing to the conversion to amorphous form and increased drug wettability in the hydrophilic polymeric matrix [7, 8]. Dukeck et al. [9] also prepared an amorphous solid dispersion of TMS using different polymers by melt quenching and showed the enhanced dissolution rate and physical stability (time to drug recrystallization) of TMS. The onset of recrystallization of the amorphous suspension of TMS (4 h) was prolonged by using different polymers, such as polyvinyl pyrrolidone (24 h), hydroxypropyl methylcellulose (48 h), Soluplus (72 h), and Eudragit (96 h). Second, the well-balanced SMEDDS formulation provided a better environment for continuous dissolution of hydrophobic drugs. Owing to the homogenously dispersed oil droplets, in which the drug exists in molecular solubilization, pertinent dissolution could take place. In addition, the presence of surfactants in the formulation further improves the BA by affecting drug absorption [16, 29]. The nano- or micro-sized emulsion droplets could improve drug dissolution rate in a pH-independent manner, in which the amount of drug dissolved in the aqueous phase at any time was inversely proportional to the radius of the droplets and the partition coefficient of the drug between the oil and aqueous phases [15, 27]. Thus, we concluded that phospholipid complex formation and SMEDDS formulation could enhance TMS dissolution in a pH-independent manner. In addition, the optimized TPC-loaded SMEDDS might be a good candidate for alternative product development in the future. Including the oral dosage forms of soft capsules, solidified forms in hard capsule are under consideration. However, further studies are still needed to assess the *in vivo* bioavailability and/or pharmacokinetic profiles.

## Conclusion

The solubility of TMS was drastically enhanced by its phospholipid complex formation (TPC). Moreover, a formulation study on TPC-loaded SMEDDS was successfully conducted using the D-optimal mixture design. The optimized TPC-loaded SMEDDS formulation, consisting of 14% Capryol 90 (oil; X_1_), 59.9% tween 80 (surfactant; X_2_), and 26.1% tetraglycol (cosurfactant; X_3_), showed an improved solubility and dissolution rate, compared to that of the raw TMS. Good agreement was found between model prediction and experimental values of droplet size (Y_1_), TMS solubilization (Y_2_), and the percentage of drug released in 15 min (Y_3_), used as the response factors. Thus, phospholipid complex formation and SMEDDS formulation using an experimental design method might be a promising approach to enhance the dissolution of poorly water-soluble drugs. For further practical development in future, *in vivo* bioavailability assessments of the optimized TPC-loaded SMEDDS would be needed.

## Supporting information

S1 Fig (A) Ternary phase diagram of Capmul MCM (oil), tween 80 (surfactant), and tetraglycol (cosurfactant). (B) Ternary phase diagram of Capryol 90 (oil), tween 80 (surfactant), and tetraglycol (cosurfactant). Light gray and dark gray areas indicate self-microemulsifying regions and experimental regions, respectively.
(TIF)

S1 Table Droplet size and PDI value of the formulation composed of Capryol 90 (oil), Tween 80 (surfactant), and Tetraglycol (cosurfactant). (DOCX)

## References

[1] Yusuf S, Diener HC, Sacco RL, Cotton D, Ôunpuu S, Lawton WA, et al Telmisartan to prevent recurrent stroke and cardiovascular events. N. Engl. J. Med. 2008 8 27; 359(12):1225–37. 10.1056/NEJMoa0804593 18753639 PMC2714258

[2] Tran PHL, Tran HTT, Lee BJ. Modulation of microenvironmental pH and crystallinity of ionizable telmisartan using alkalizers in solid dispersions for controlled release. J. Control. Release. 2008 4 13; 129(1):59–65. 10.1016/j.jconrel.2008.04.001 18501462

[3] Wienen W, Entzeroth M, van Meel JC, Stangier J, Busch U, Ebner T, et al A review on telmisartan: a novel, long-acting angiotensin II-receptor antagonist. Cardiovasc. Drug Rev. 2008 6 7; 18(2):127–54. 10.1111/j.1527-3466.2000.tb00039.x

[4] Zhang Y, Zhi Z, Jiang T, Zhang J, Wang Z, Wang S. Spherical mesoporous silica nanoparticles for loading and release of the poorly water-soluble drug telmisartan. J. Control. release. 2010 5 5; 145(3):257–63. 10.1016/j.jconrel.2010.04.029 20450945

[5] Cho HJ, Lee DW, Marasini N, Poudel BK, Kim JH, Ramasamy T, et al Optimization of self-microemulsifying drug delivery system for telmisartan using Box–Behnken design and desirability function. J.Pharm. Pharmacol. 2013 7 24; 65(10):1440–50. 10.1111/jphp.12115 24028611

[6] Konno H, Handa T, Alonzo DE, Taylor LS. Effect of polymer type on the dissolution profile of amorphous solid dispersions containing felodipine. Eur. J. Pharm. Biopharm. 2008 6 6; 70(2):493–9. 10.1016/j.ejpb.2008.05.023 18577451

[7] Kothawade SN, Kadam NR, Aragade PD, Baheti DG. Formulation and characterization of telmisartan solid dispersion. Int. J. PharmTech Res. 2010 11 19; 2:341–7.

[8] Patel B, Parikh RH, Swarnkar D. Enhancement of dissolution of telmisartan through use of solid dispersion technique–surface solid dispersion. J. Pharm. BioAllied Sci. 2012 3 4; 4(Suppl 1):64–8. 10.4103/0975-7406.94142 23066211 PMC3467836

[9] Dukeck R, Sieger P, Karmwar P. Investigation and correlation of physical stability, dissolution behaviour and interaction parameter of amorphous solid dispersions of telmisartan: A drug development perspective. Eur. J. Pharm. Sci. 2013 5 16; 49(4):723–31. 10.1016/j.ejps.2013.05.003 23684913

[10] Yamashita S, Fukunishi A, Higashino H, Kataoka M, Wada K. Design of supersaturable formulation of telmisartan with pH modifier: in vitro study on dissolution and precipitation. J. Pharm. Invest. 2017 3; 47(2):163–71. 10.1007/s40005-017-0310-3

[11] Venema FR, Weringa WD. The interactions of phospholipid vesicles with some anti-inflammatory agents. J. Colloid Interface Sci. 1988 10; 125(2):484–500. 10.1016/0021-9797(88)90013-6

[12] Maiti K, Mukherjee K, Gantait A, Saha BP, Mukherjee PK. Curcumin–phospholipid complex: Preparation, therapeutic evaluation and pharmacokinetic study in rats. Int. J. Pharm. 2007 9 23; 330(1–2):155–63. 10.1016/j.ijpharm.2006.09.025 17112692

[13] Zhang J, Peng Q, Shi S, Zhang Q, Sun X, Gong T, et al Preparation, characterization, and in vivo evaluation of a self-nanoemulsifying drug delivery system (SNEDDS) loaded with morin-phospholipid complex. Int. J. Nanomed. 2011 12 19; 6:3405–14. 10.2147/IJN.S25824 22267925 PMC3260034

[14] Cui F, Shi K, Zhang L, Tao A, Kawashima Y. Biodegradable nanoparticles loaded with insulin–phospholipid complex for oral delivery: Preparation, in vitro characterization and in vivo evaluation. J. Control. Release. 2006 8 28; 114(2):242–50. 10.1016/j.jconrel.2006.05.013 16859800

[15] Ahmad J, Kohli K, Mir SR, Amin S. Formulation of self-nanoemulsifying drug delivery system for telmisartan with improved dissolution and bioavailability. J. Dispersion Sci. Technol. 2011 6 27; 32(7):958–68. 10.1080/01932691.2010.488511

[16] Jaiswal P, Aggarwal G, Harikumar SL, Singh K. Development of self-microemulsifying drug delivery system and solid-self-microemulsifying drug delivery system of telmisartan. Int. J. Pharm. Invest. 2014 10; 4(4):195–206. 10.4103/2230-973X.143123 25426441 PMC4241625

[17] Marasini N, Tran TH, Poudel BK, Cho HJ, Choi YK, Chi SC, et al Fabrication and evaluation of pH-modulated solid dispersion for telmisartan by spray-drying technique. Int. J. Pharm. 2013 11 19; 441(1–2):424–32. 10.1016/j.ijpharm.2012.11.012 23174408

[18] Yang L, Shao Y, Han HK. Improved pH-dependent drug release and oral exposure of telmisartan, a poorly soluble drug through the formation of drug-aminoclay complex. Int. J. Pharm. 2014 5 14; 471(1–2):258–63. 10.1016/j.ijpharm.2014.05.009 24834880

[19] Bruning T, Bolt HM. Renal toxicity and carcinogenicity of trichloroethylene: key results, mechanisms, and controversies. Crit. Rev. Toxicol. 2000 5; 30(3):253–85. 10.1080/10408440091159202 10852497

[20] Vasconcelos T, Sarmento B, Costa P. Solid dispersions as strategy to improve oral bioavailability of poor water soluble drugs. Drug Discovery Today. 2007 10 30; 12(23–24):1068–75. 10.1016/j.drudis.2007.09.005 18061887

[21] Khoo SM, Humberstone AJ, Porter CJ, Edwards GA, Charman WN. Formulation design and bioavailability assessment of lipidic self-emulsifying formulations of halofantrine. Int. J. Pharm. 1998 6; 167(1–2):155–64. 10.1016/S0378-5173(98)00054-4

[22] Holm R, Jensen IHM, Sonnergaard J. Optimization of self-microemulsifying drug delivery systems (SMEDDS) using a D-optimal design and the desirability function. Drug Dev. Ind. Pharm. 2006 10; 32(9):1025–32. 10.1080/03639040600559024 17012115

[23] Gu B, Burgess DJ. Prediction of dexamethasone release from PLGA microspheres prepared with polymer blends using a design of experiment approach. Int. J. Pharm. 2015 9 15; 495(1):393–403. 10.1016/j.ijpharm.2015.08.089 26325309 PMC4609624

[24] Tran T, Rades T, Müllertz A. Formulation of self-nanoemulsifying drug delivery systems containing monoacyl phosphatidylcholine and Kolliphor RH40 using experimental design. Asian J. Pharm. Sci. 2017 9; 10.1016/j.ajps.2017.09.006 PMC7037638 32104428

[25] Liu Y, Zhang P, Feng N, Zhang X, Wu S, Zhao J. Optimization and in situ intestinal absorption of self-microemulsifying drug delivery system of oridonin. Int. J. Pharm. 2009 8 20; 365(1–2):136–42. 10.1016/j.ijpharm.2008.08.009 18782611

[26] Marasini N, Yan YD, Poudel BK. Development and optimization of self-nanoemulsifying drug delivery system with enhanced bioavailability by Box–Behnken design and desirability function. J. Pharm. Sci. 2012 9 28; 101(12):4584–96. 10.1002/jps.23333 23023800

[27] Yeom DW, Chae BR, Son HY, Kim JH, Chae JS, Song SH, et al Enhanced oral bioavailability of valsartan using a polymer-based supersaturable self-microemulsifying drug delivery system. Int. J. Nanomed. 2017 5 8; 12:3533–45. 10.2147/IJN.S136599 28507434 PMC5428796

[28] Mura P, Furlanetto S, Cirri M, Maestrelli F, Marras AM, Pinzauti S. Optimization of glibenclamide tablet composition through the combined use of differential scanning calorimetry and D-optimal mixture experimental design. J. Pharm. Biomed. Anal. 2005 2 7; 37(1):65–71. 10.1016/j.jpba.2004.09.047 15664744

[29] Yeom DW, Song YS, KIM SR, Lee SG, Kang MH, Lee SK, et al Development and optimization of a self-microemulsifying drug delivery system for atorvastatin calcium by using D-optimal mixture design. Int. J. Nanomed. 2015 6 5; 10:3865–78. 10.2147/IJN.S83520 26089663 PMC4462857

[30] Chae JS, Chae BR, Shin DJ, Goo YT, Lee ES, Yoon HY, et al Tablet formulation of a polymeric solid dispersion containing amorphous alkalinized telmisartan. AAPS PharmSciTech. 2018 7 24; Accepted. 10.1208/s12249-018-1124-y 30043191

[31] O’Neil MJ. The Merck Index: An encyclopedia of chemicals, drugs, and biologicals. 15th ed Cambridge, UK: Royal Society of Chemistry; 2013 pp. 1691.

[32] Yanyu X, Yunmei S, Zhipeng C, Qineng P. The preparation of silybin–phospholipid complex and the study on its pharmacokinetics in rats. Int. J. Pharm. 2006 1 3; 307(1):77–82. 10.1016/j.ijpharm.2005.10.001 16300915

[33] Ruan J, Liu J, Zhu D, Gong T, Yang F, Hao X, et al Preparation and evaluation of self-nanoemulsified drug delivery systems (SNEDDSs) of matrine based on drug-phospholipid complex technique. Int. J. Pharm. 2010 2 15; 386(1–2):282–90. 10.1016/j.ijpharm.2009.11.026 19961910

[34] Kang BK, Lee JS, Chon SK, Jeong SY, Yuk SH, Khang G, et al Development of self-microemulsifying drug delivery systems (SMEDDS) for oral bioavailability enhancement of simvastatin in beagle dogs. Int. J. Pharm. 2004 4 15; 274(1–2):65–73. 10.1016/j.ijpharm.2003.12.028 15072783

[35] Oh DJ, Kang JH, Kim DW, Lee BJ, Kim JO, Yong CS, et al Comparison of solid self-microemulsifying drug delivery system (solid SMEDDS) prepared with hydrophilic and hydrophobic solid carrier. Int. J. Pharm. 2011 9 16; 420(2):412–8. 10.1016/j.ijpharm.2011.09.007 21944892

[36] Padia N, Shukla A, Shelat P. Development and characterization of fenofibrate self-microemulsifying drug delivery system (SMEDDS) for bioavailability enhancement. Bull. Pharm. Res. 2015 8; 5(2):59–69.

[37] Liu Y, Sun C, Hao Y, Jiang T, Zheng L, Wang S. Mechanism of dissolution enhancement and bioavailability of poorly water soluble celecoxib by preparing stable amorphous nanoparticles. J. Pharm. Pharm. Sci. 2010; 13(4):589–606. 10.18433/J3530J 21486533

