# Supplementary figures and images for: Optimization of self-microemulsifying drug delivery system for phospholipid complex of telmisartan using D-optimal mixture design
Source: PLoS One. 2018 Dec 5;13(12):e0208339. doi: 10.1371/journal.pone.0208339 (PMC6281252; doi:10.1371/journal.pone.0208339)

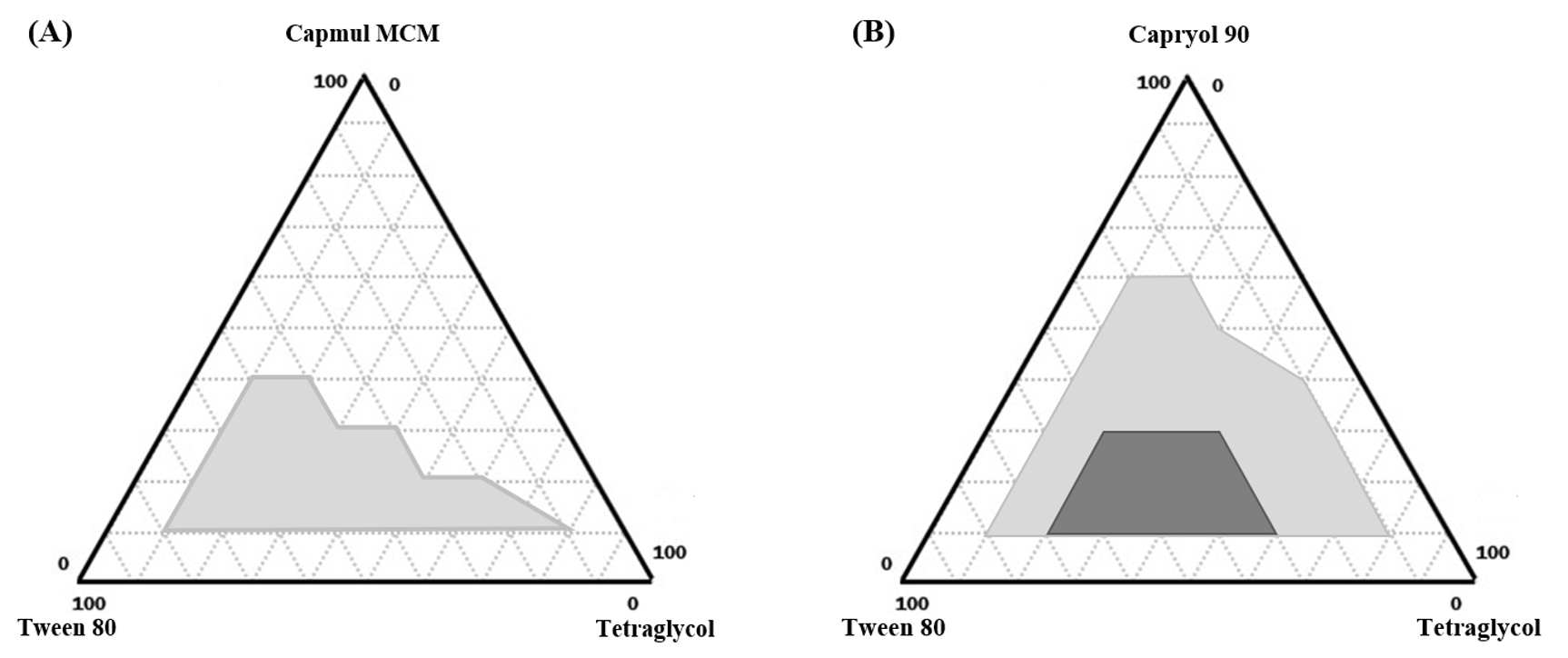

Supplement: S1 Fig — (A) Ternary phase diagram of Capmul MCM (oil), tween 80 (surfactant), and tetraglycol (cosurfactant). (B) Ternary phase diagram of Capryol 90 (oil), tween 80 (surfactant), and tetraglycol (cosurfactant). Light gray and dark gray areas indicate self-microemulsifying regions and experimental regions, respectively. (TIF) [file pone.0208339.s001.tif]
