# Supplementary material for: Optimization of self-microemulsifying drug delivery system for phospholipid complex of telmisartan using D-optimal mixture design
Source: PLoS One. 2018 Dec 5;13(12):e0208339. doi: 10.1371/journal.pone.0208339 (PMC6281252; doi:10.1371/journal.pone.0208339)
Supplement: S1 Table — (DOCX) [file pone.0208339.s002.docx]

| **S1 Table**. Droplet size data of Capryol 90 (oil), Tween 80 (surfactant), and Tetraglycol (cosurfactant) | | | | |
| --- | --- | --- | --- | --- |
| **Capryol 90 (%)** | **Tween 80 (%)** | **Tetraglycol (%)** | **Droplet size (nm)** | **PDI^*^** |
| 10 | 80 | 10 | 12.9 ± 0.1 | 0.08 ± 0.01 |
| 10 | 70 | 20 | 13.7 ± 0.1 | 0.09 ± 0.04 |
| 10 | 60 | 30 | 14.9 ± 0.1 | 0.10 ± 0.01 |
| 10 | 50 | 40 | 15.8 ± 0.2 | 0.12 ± 0.03 |
| 10 | 40 | 50 | 17.5 ± 0.2 | 0.17 ± 0.01 |
| 10 | 30 | 60 | 40.0 ± 0.3 | 0.29 ± 0.01 |
| 10 | 20 | 70 | 86.8 ± 0.6 | 0.30 ± 0.03 |
| 10 | 10 | 80 | 92.4 ± 2.8 | 0.35 ± 0.03 |
| 20 | 70 | 10 | 131.4 ± 2.7 | 0.48 ± 0.01 |
| 20 | 60 | 20 | 120.2 ± 3.4 | 0.28 ± 0.00 |
| 20 | 50 | 30 | 118.7 ± 0.5 | 0.29 ± 0.02 |
| 20 | 40 | 40 | 129.9 ± 2.0 | 0.29 ± 0.01 |
| 20 | 30 | 50 | 128.9 ± 5.1 | 0.29 ± 0.01 |
| 20 | 20 | 60 | 126.9 ± 2.7 | 0.39 ± 0.01 |
| 20 | 10 | 70 | 136.4 ± 4.3 | 0.19 ± 0.03 |
| 30 | 60 | 10 | 150.8 ± 3.0 | 0.31 ± 0.03 |
| 30 | 50 | 20 | 138.6 ± 2.6 | 0.29 ± 0.01 |
| 30 | 40 | 30 | 144.3 ± 8.2 | 0.29 ± 0.00 |
| 30 | 30 | 40 | 131.4 ± 3.3 | 0.28 ± 0.00 |
| 30 | 20 | 50 | 146.5 ± 14.7 | 0.31 ± 0.12 |
| 30 | 10 | 60 | 149.7 ± 8.8 | 0.24 ± 0.01 |
| 40 | 50 | 10 | 179.9 ± 2.2 | 0.36 ± 0.04 |
| 40 | 40 | 20 | 194.5 ± 5.9 | 0.47 ± 0.02 |
| 40 | 30 | 30 | 163.9 ± 3.9 | 0.34 ± 0.06 |
| 40 | 20 | 40 | 139.3 ± 1.9 | 0.27 ± 0.03 |
| 40 | 10 | 50 | 139.2 ± 4.7 | 0.27 ± 0.03 |
| 50 | 40 | 10 | 154.4 ± 4.9 | 0.46 ± 0.04 |
| 50 | 30 | 20 | 150.6 ± 7.7 | 0.39 ± 0.01 |
| 50 | 20 | 30 | 150.7 ± 8.8 | 0.34 ± 0.01 |
| 50 | 10 | 40 | 150.3 ± 4.0 | 0.29 ± 0.05 |
| 60 | 30 | 10 | 159.1 ± 2.4 | 0.27 ± 0.02 |
| 60 | 20 | 20 | 154.6 ± 4.3 | 0.24 ± 0.06 |
| 60 | 10 | 30 | 154.9 ± 0.7 | 0.35 ± 0.04 |
| 70 | 20 | 10 | 156.4 ± 1.8 | 0.38 ± 0.04 |
| 70 | 10 | 20 | 170.6 ± 7.3 | 0.22 ± 0.11 |
| 80 | 10 | 10 | 198.9 ± 8.1 | 0.38 ± 0.02 |
| ^*^PDI, Polydispersity index | |  |  |  |
